# Editorial: Methods and protocols in Brain-Computer Interfaces

**DOI:** 10.3389/fnhum.2024.1447973

**Published:** 2024-07-17

**Authors:** Anastassia Angelopoulou, Ines Chihi, Jude Hemanth

**Affiliations:** ^1^Department of Computer Science, University of Westminster, London, United Kingdom; ^2^Department of Engineering, University of Luxembourg, Luxembourg, United Kingdom; ^3^Department of Electronics and Communication Engineering, Karunya Institute of Technology and Sciences, Coimbatore, Tamil Nadu, India

**Keywords:** Brain-Computer Interface (BCI), frameworks and architectures, EEG, methods and algorithms, design methodology

With rapid progress of technology, research on Brain-Computer Interfaces (BCIs) assistive devices has experienced abundant growth over the last two decades and its importance and position has been proved and influenced by many scientists, professionals and researchers in many applications such as electronics, physics, biochemistry, signal and image processing, integrated sensors-actuators and more. Both invasive and non-invasive BCIs have demonstrated the possibility of decoding motor control parameters obtained from different sources (*e.g., electroencephalography (EEG), magnetoencephalography (MEG), etc*.) that involves kinematics, movement and grasping patterns.

BCIs have made significant progress in enabling human subjects to operate wearable devices, by measures of neural and physiological activity, in a continuous and independent way. Notwithstanding these advances, several challenges need to be overcome for these systems to be ready for use in different environments (*e.g., medical applications, entertainment industry, etc*.) ranging from stable and robust performance to continuous adaptation to user/system requirements (Millán et al., 2010; Soekadar et al., 2016; Simon et al., 2021; Catalán et al., 2023).

This Research Topic is part of the Methods and Protocols in Human Neuroscience series and aims to promote the latest experimental techniques and methods used to investigate fundamental questions in BCIs research. Each paper was reviewed by at least two reviewers with two rounds before acceptance after a rigorous revision process. Three articles were selected for original research and one review article.

Listed below are the papers that made important contributions to this discussion.

Schmoigl-Tonis et al. conducted a systematic literature review on devices, software tools, methods and algorithms on motion artifact reduction in BCIs with the aim to create a comprehensive lookup table for the community to facilitate comparison and analysis of existing architectures and methods. Their findings identified limitations on sample sizes of BCI, data comparison across multiple studies, a gap in studies addressing the ground truth problem, bias toward newly introduced pipelines/methods over existing ones, and finally datasets to be challenging due to variations in paradigms, participant introductions, recording environments, hardware setups and preprocessing steps. Based on the above findings, the authors emphasize the need for further research in motion artifact reduction in BCI experiments in order to provide valuable insights for researchers and practitioners.

Leeuwis et al. performed an extensive study, by examining the difference between High and Low aptitude BCIs users/performers in a Motor Imagery (MI) task, with the aim to establish the relationship between EEG functional connectivity in recognizing BCI inefficient users. The study was conducted on a dataset collected by the authors and their analysis includes three network scales (Global, Large and Local scale) during the resting-state, left vs. right-hand motor imagery task, and the transition between the two phases. They reported that functional connectivity might be a valuable feature in MI-BCI classification and in solving the MI-BCI inefficiency problem.

Almajidy et al. developed a hybrid BCI system with one optical and two electrical modalities for the implementation of a two-dimensional motor imagery paradigm in off- and online sessions to sixteen volunteers. The novel hardware consisted of a near-infrared spectroscopy (NIRS) device integrated with an EEG system that used two different types of electrodes. Their approach effectively demonstrates improvement in classification accuracy when using tri-polar concentric ring electrodes (TCRE) with EEG. Their findings underscores the effectiveness of this modular system to monitor brain activity at different regions of interest in an affordable, portable, and lightweight manner.

Gemborn Nilsson et al. study demonstrates a significant contribution to addresses limitations in current BCIs, offering improvements for real-time classification and decoding of user states. The main contribution aims introducing an open-source research framework that enables human-in-the-loop model training, real-time stimulus control, and online EEG data classification. The framework supports offline EEG data analysis and discusses desirable properties for BCI research platforms. Overall, it significantly enhances the capabilities and accessibility of BCIs in various fields, ranging from medical applications to entertainment industry, and creates new conditions for basic research in cognitive neuroscience.

In conclusion, we express our gratitude to the authors and reviewers who contributed to this special Research Topic on Methods and Protocols in Brain-Computer Interfaces (BCIs). The high-quality papers in this Research Topic demonstrate the capabilities of researchers in advancing knowledge and innovation in systems, methodologies, frameworks and applications relevant to BCIs.

## Author contributions

AA: Conceptualization, Writing – original draft, Writing – review & editing. IC: Writing – original draft, Writing – review & editing. JH: Writing – review & editing, Writing – original draft.
